# Acute Hemianopia Associated With Nonketotic Hyperglycemia: A Case Report With Advanced Neuroimaging Findings

**DOI:** 10.7759/cureus.84708

**Published:** 2025-05-23

**Authors:** Honami Tanifuji, Kouhei Kamiya, Genki Sato, Arisa Yoshida, Masaaki Hori

**Affiliations:** 1 Department of Radiology, Toho University Graduate School of Medicine, Tokyo, JPN; 2 Department of Diabetes,Metabolism and Endcrinology, Toho University Graduate School of Medicine, Tokyo, JPN

**Keywords:** arterial spin labeling (asl), magnetic resonance spectroscopy (mrs), nonketotic hyperglycemia, single-photon emission computed tomography, t2-weighted imaging, visual field disorder

## Abstract

Nonketotic hyperglycemia (NKH) can present with acute neurological symptoms such as tremor, ballismus, seizures, hemianopia, and coma, and may be associated with abnormal signal intensities on brain MRI. We report the case of a woman in her 50s with poorly controlled diabetes mellitus (HbA1c 15.9%) who was admitted with a chief complaint of right homonymous hemianopia. No other neurological deficits were observed. At presentation, the patient's blood glucose level was markedly elevated (608 mg/dL), with a blood pH of 7.36, negative ketone bodies, and an increased serum osmolality of 301 mOsm/L, consistent with a diagnosis of nonketotic hyperglycemia (NKH). Brain MRI performed on the day of admission revealed subcortical hypointensities in the left occipital and parietal lobes on T2-weighted images (T2WI). Arterial spin labeling (ASL) demonstrated increased perfusion in the corresponding areas. Proton magnetic resonance spectroscopy (¹H-MRS) showed a reduction in N-acetylaspartate (NAA) levels in the same region. Following glycemic control, her visual field deficits improved, and the subcortical MRI abnormalities resolved. Given the temporal correlation between symptom resolution and imaging normalization, the patient was diagnosed with NKH-induced hemianopia with transient MRI abnormalities. While subcortical T2 hypointensities are a known radiologic feature of NKH, associated findings on ASL and ¹H-MRS have been rarely documented. We present this case alongside a review of the relevant literature.

## Introduction

Nonketotic hyperglycemia (NKH) is a serious metabolic complication typically associated with poorly controlled type 2 diabetes mellitus. It is characterized by severe hyperglycemia, hyperosmolarity, and intracellular dehydration, occurring with little or no ketoacidosis [1]. Although NKH can occur in both type 1 and type 2 diabetes, it is more commonly seen in patients with type 2 diabetes. Neurological manifestations of NKH may include chorea, ballismus, seizures, coma, and visual field disturbances. Visual symptoms may present as visual hallucinations, photopsia, blurred vision, or homonymous hemianopia. MRI findings in patients with NKH-related visual symptoms may reveal subcortical hypointensity on T2-weighted imaging (T2WI), often accompanied by hyperintensity in the overlying cortex [2]. These imaging abnormalities are frequently transient and tend to resolve following appropriate glycemic control [3,4].

Particularly, homonymous hemianopia is an extremely rare symptom in NKH, and its clinical significance remains largely unclear. Although it is suggested that localized perfusion abnormalities and neuro-metabolic changes in the occipital lobe may be involved, the scarcity of case reports leaves its role in diagnosis and prognostic prediction uncertain. Nevertheless, the manifestation of homonymous hemianopia is believed to reflect a direct impact of hyperglycemia on the occipital lobe, and specific neuroimaging findings can capture this pathophysiological process. Therefore, detailed imaging analysis of cases presenting with homonymous hemianopia could serve as a crucial clue to understanding the pathophysiology of NKH.

In this report, we present a case of NKH complicated by acute visual field defects and subcortical T2 hypointensity in the occipital lobe. Given the limited literature on ASL and proton magnetic resonance spectroscopy (¹H-MRS) in similar cases, we highlight the advanced neuroimaging findings observed in this patient.

## Case presentation

The patient was a 53-year-old woman with a medical history significant for type 1 diabetes mellitus and chronic renal failure, for which she had been undergoing hemodialysis since the age of 38. On Day 0, she was aware of right homonymous hemianopia. This symptom progressively worsened, and by Day 6, the right hemifield had become completely obscured, resulting in marked visual impairment. She presented to the emergency department on the same day.

Upon arrival, her vital signs were largely within normal limits, with the exception of mild tachycardia. In the confrontation visual field test, right homonymous hemianopia was observed, though no other gross neurological abnormalities were noted. Laboratory tests (Table 1) revealed elevated serum creatinine (6.49 mg/dL), marked hyperglycemia (608 mg/dL), hyponatremia (129 mmol/L), and a significantly elevated HbA1c of 15.9%. The patient was also hyperosmolar (301 mOsm/L), with a blood pH of 7.36 and negative blood ketones, supporting a diagnosis of nonketotic hyperglycemia. Urinalysis could not be performed due to her dialysis-dependent status.

**Table 1:** Comprehensive laboratory findings at presentation

Parameter	Patient Value	Reference Range
White Blood Cell Count (×10⁹/L)	4.5	4.0–10.0
Red Blood Cell Count (×10⁹/L)	3.66	4.5–5.9 (M), 4.1–5.1 (F)
Hemoglobin (g/dL)	11.4	13.0–17.0 (M), 12.0–15.0 (F)
Serum Creatinine (mg/dL)	6.49	0.6–1.2
Blood Urea Nitrogen (mg/dL)	24	7–20
Estimated Glomerular Filtration Rate (mL/min/1.73m²)	5.9	>90
Alanine Aminotransferase (U/L)	9	7–55
Aspartate Aminotransferase (U/L)	13	8–48
Sodium (Na⁺) (mmol/L)	129	135–145
Potassium (K⁺) (mmol/L)	4.5	3.5–5.1
Serum Osmolality (mOsm/L)	301	275-290
Glucose (mg/dL)	608	70–110
D-dimer (µg/mL)	1.3	< 0.5
Glycated Hemoglobin (HbA1c) (%)	15.9	< 5.7
pH	7.366	7.35–7.45
Partial Pressure of Oxygen (PaO₂) (mmHg)	122	80–100
Partial Pressure of Carbon Dioxide (PaCO₂) (mmHg)	38.8	35–45
Bicarbonate (HCO₃⁻) (mmol/L)	21.7	22–28
Base Excess (mmol/L)	-2.7	-2.0–+2.0

Ophthalmological evaluation revealed right homonymous hemianopia. Fundoscopic examination demonstrated diabetic retinopathy with associated retinal hemorrhages; however, no evidence of vitreous hemorrhage was observed. A non-contrast head CT scan demonstrated a mottled hypoattenuating area in the left cerebellar hemisphere. No other abnormalities were identified that could account for the patient’s visual symptoms.

**Figure 1:**
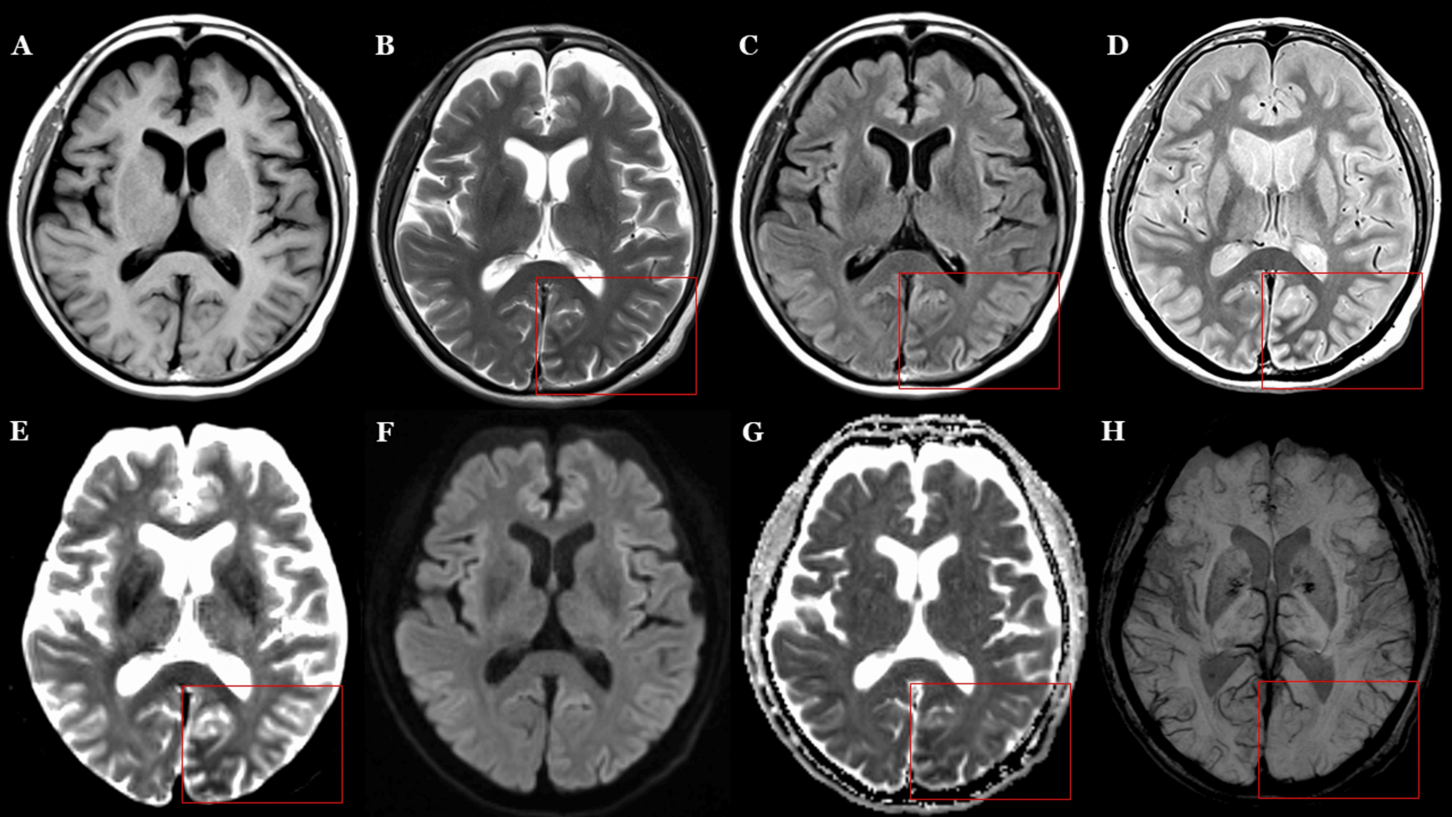
Head MRI on day 2 of admission (day 7 of right homonymous hemianopia onset) shows an area of abnormal signal (red frames) No abnormal findings are observed on T1-weighted imaging (A). Subcortical hypointensity in the left occipital lobe is observed on T2-weighted imaging (B), FLAIR (C), proton density weighted imaging (D), b=0 (E), and ADC map (G). No abnormalities are noted on DWI with a b-value of 1000s/mm² (F). On SWI (H), decreased visibility of veins in the left occipital lobe is noted, which may reflect increased regional perfusion. FLAIR: fluid-attenuated inversion recovery; ADC: apparent diffusion coefficient; DWI: diffusion-weighted imaging; SWI: susceptibility-weighted imaging

**Figure 2:**
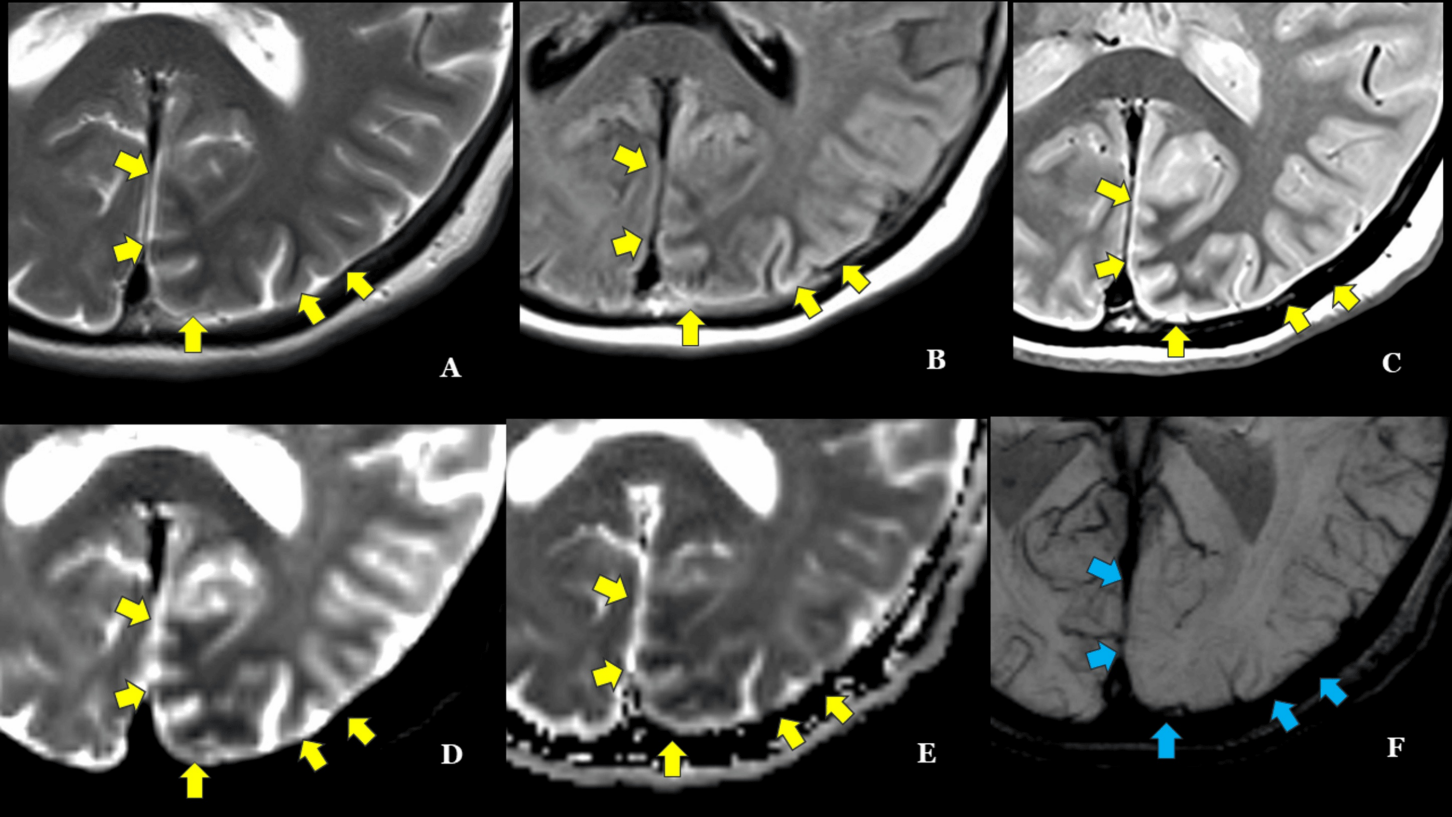
Magnified image of the lesion observed on MRI in Figure 1 (A) T2-weighted imaging, (B) FLAIR, (C) proton-density weighted imaging, (D) b=0, and (E) ADC map; hypointensity is observed in the subcortical white matter (yellow arrows). (F) SWI showing decreased venous visibility (blue arrow). FLAIR: fluid-attenuated inversion recovery; ADC: apparent diffusion coefficient; SWI: susceptibility-weighted imaging

**Figure 3:**
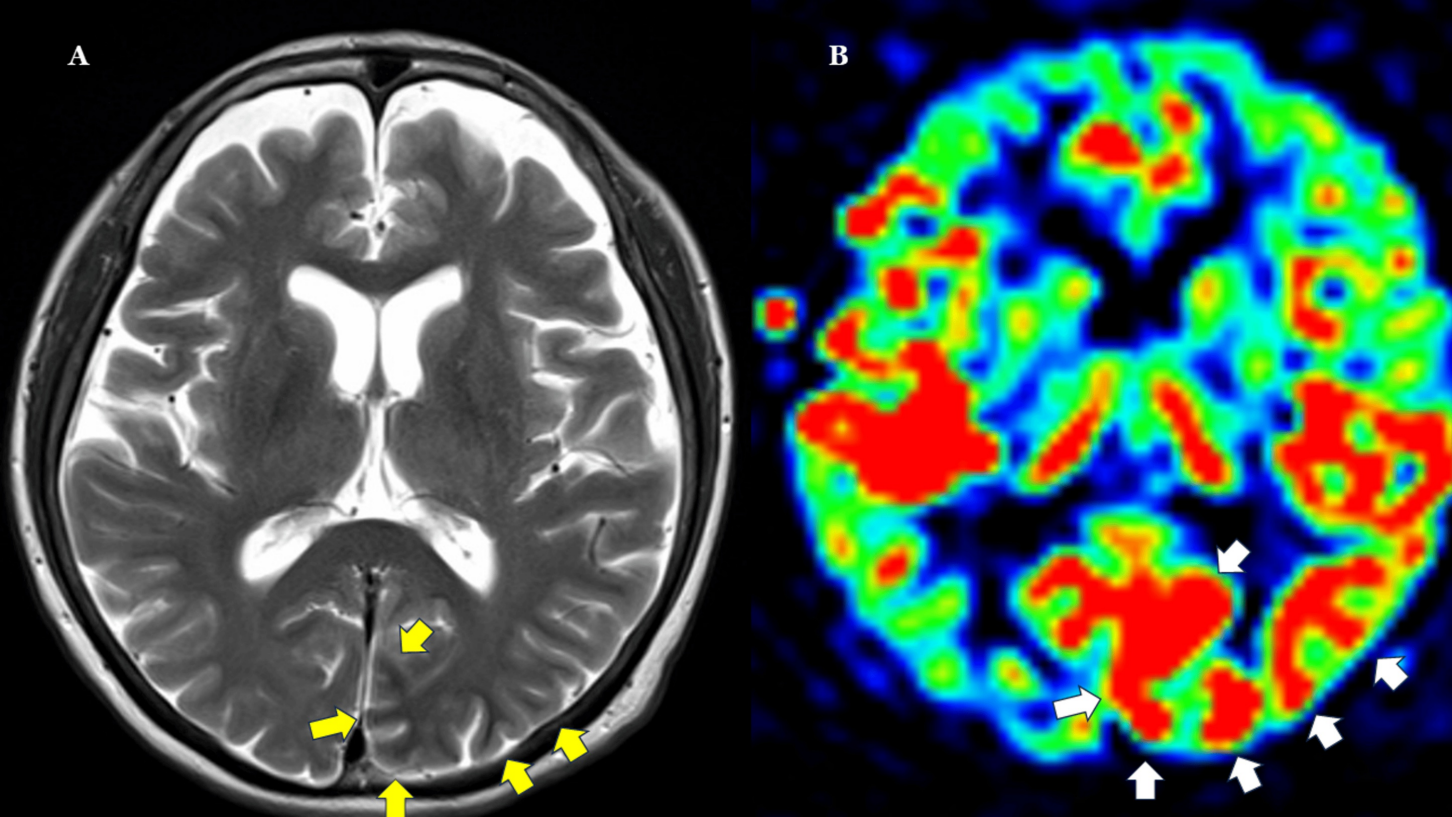
ASL on day 2 of admission (day 7 of right homonymous hemianopia onset) On ASL with a bolus duration of 700 ms and an inversion time of 1990 ms, increased perfusion is observed in the cortical region of the left occipital lobe (white arrow) on the color-coded CBF map (B), corresponding to the subcortical hypointense area (yellow arrow) on T2-weighted imaging (A). ASL: arterial spin labeling; CBF: cerebral blood flow

On the day following admission (day 7 of right homonymous hemianopia), brain MRI revealed subcortical hypointensity on T2WI images, predominantly involving the left occipital lobe. Susceptibility-weighted imaging (SWI) demonstrated diminished venous visibility in the same region. No abnormalities were observed on diffusion-weighted imaging or apparent diffusion coefficient (ADC) maps. Magnetic resonance angiography(MRA) showed no significant asymmetry in the major cerebral arteries, including the posterior cerebral arteries (Figure 1). Enlarged views of the lesion within the red frames shown in Figure 1 are presented in Figure 2. Arterial spin labeling (ASL) perfusion imaging demonstrated increased cerebral blood flow (CBF) in the left occipito-parietal region (Figure 3).

Clinical course

**Figure 4:**
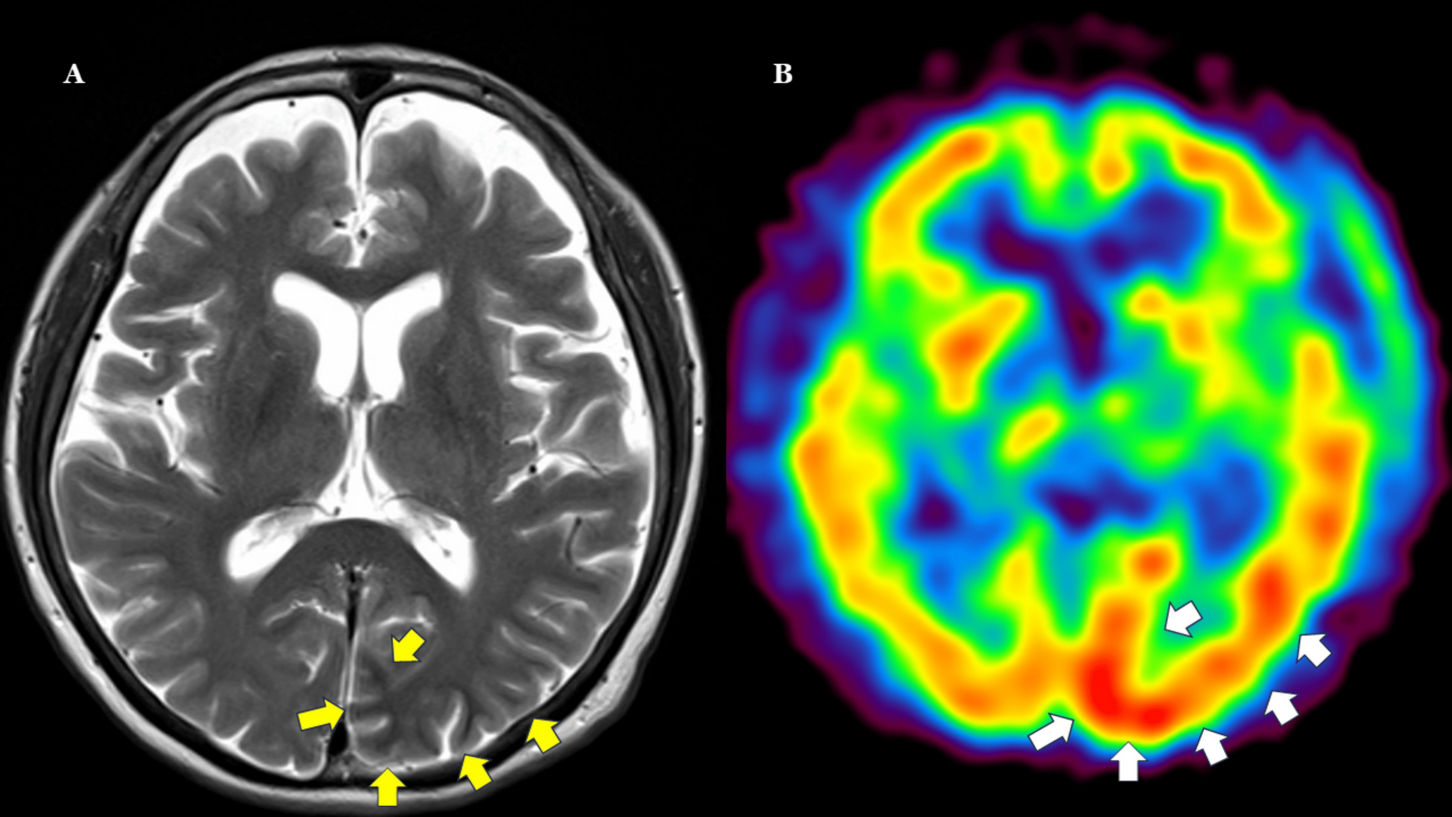
¹²³I-IMP SPECT on day 10 of right homonymous hemianopia onset in comparison with T2-weighted MRI on day 7. On ¹²³I-IMP SPECT (B), increased accumulation is observed in the cortical region of the left occipital lobe (white arrow), corresponding to the hypointense area (yellow arrow) on T2WI MRI (A) obtained on Day 7. ¹²³I-IMP SPECT: N-isopropyl-p-[¹²³I]iodoamphetamine single-photon emission computed tomography

Insulin therapy was initiated on the day of admission (day 6 of right homonymous hemianopia). Electroencephalography (EEG) performed on days 8 and 11 of right homonymous hemianopia showed no epileptiform activity. On day 10 of right homonymous hemianopia, N-isopropyl-p-(^123^I)-iodoamphetamine single-photon emission CT (¹²³I-IMP SPECT) imaging (Figure 4) revealed increased tracer accumulation in the medial to dorsolateral regions of the left occipital lobe. By day 12 of right homonymous hemianopia, the patient’s subjective visual symptoms had resolved. Goldmann perimetry on day 16 of right homonymous hemianopia revealed a residual left inferior quadrantanopia, indicating an objective trend toward improvement. Subjective symptoms continued to improve thereafter, and the patient was scheduled for follow-up with an ophthalmologist after discharge.

**Figure 5:**
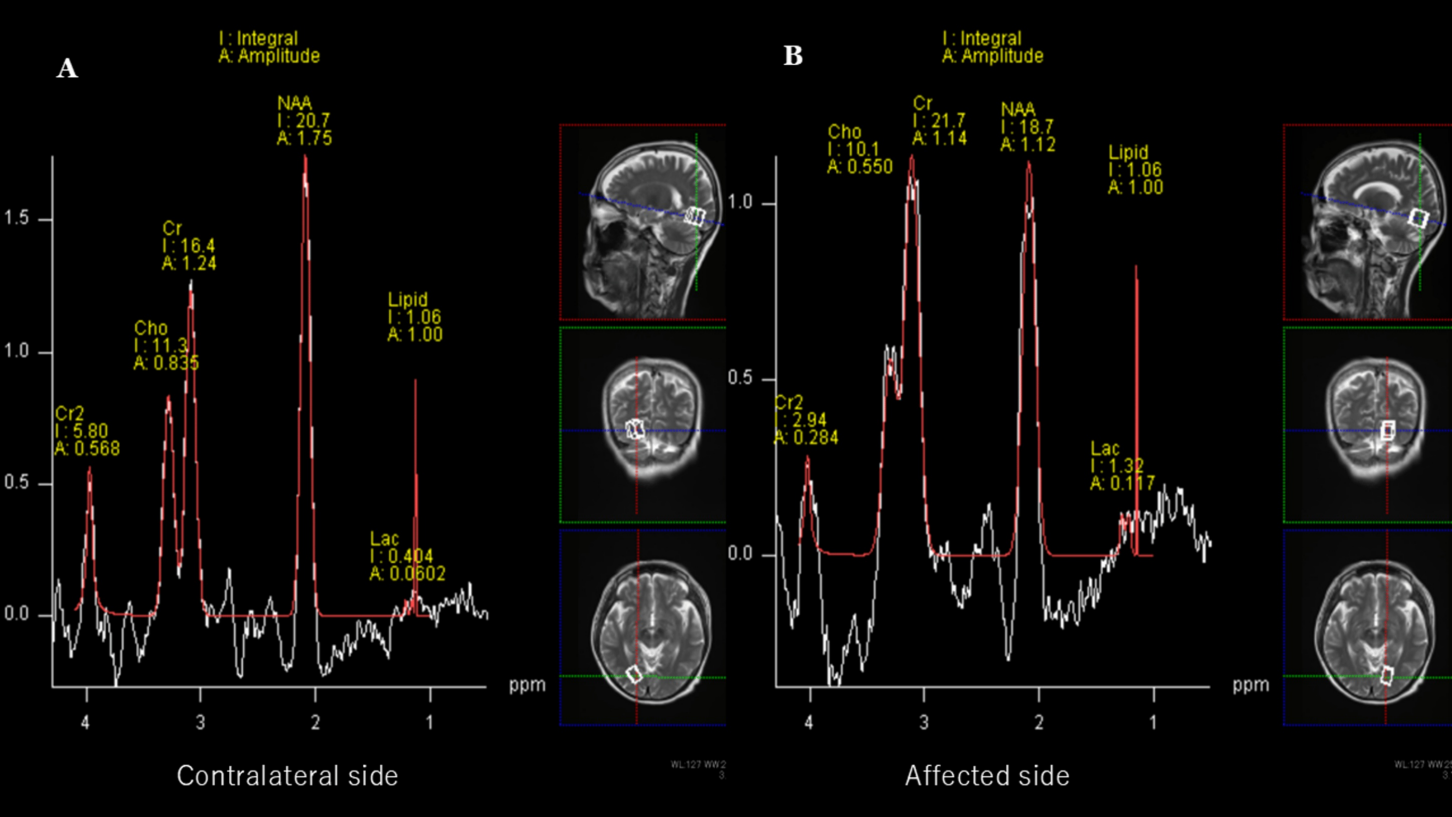
¹H-MRS on day 17 of of right homonymous hemianopia onset ¹H-MRS was performed with volumes of interest placed in the right (A) and left (B) occipital lobes. Compared to the contralateral side (A), the affected side (B) showed a decreased N-acetylaspartate to creatine ratio (NAA/Cr: 0.86 vs. 1.26), indicating reduced neuronal integrity. Additionally, the lactate to creatine ratio (Lac/Cr) was elevated on the affected side (0.060) compared to the contralateral side (0.024). ¹H-MRS: Proton magnetic resonance spectroscopy,ppm: Parts per million,NAA: N-acetylaspartate,Cr: Creatine,Lac: Lactate,Cho: Choline

¹H-MRS performed on day 17 of right homonymous hemianopia (Figure 4) showed a decreased N-acetylaspartate to creatine ratio (NAA/Cr: 0.86 vs. 1.26), and an elevated lactate peak in the affected hemisphere compared to the contralateral side (Lac/Cr: 0.060 vs. 0.024), findings suggestive of neuronal damage and increased anaerobic metabolism (Figure 5). No significant increase in choline was observed. The patient was discharged on day 27 of right homonymous hemianopia with complete resolution of her symptoms.

## Discussion

Here, we presented a case of right homonymous hemianopsia due to nonketotic hyperglycemia with transient subcortical hypointensity on T2WI. Neurological symptoms associated with NKH generally include tremor, ballismus, seizures, and coma [4]. Rarely, NKH causes transient damage to the visual cortex, resulting in homonymous hemianopia [4]. However, the mechanism remains unknown.

In our patient, MRI revealed subcortical hypointensity on T2WI in the left occipital lobe. SWI showed diminished venous visibility (presumed to reflect increased intravenous signal), and ASL and ¹²³I-IMP SPECT suggested increased perfusion in the left occipito-parietal region. Hypothetical causes of subcortical hypointensity on T2WI in NKH include intracellular dehydration due to hyperosmolar pressure, generation of reactive oxygen species, altered neurotransmitter function, mechanical changes in intracellular enzymes [1,2], and iron accumulation [1,3,5]. While a few reports have described NKH with high cortical signals on DWI [4], no abnormalities were observed on DWI or ADC maps in our patient.

Other conditions that can present with subcortical hypointensities on T2WI include intracranial hypotension, head trauma, encephalitis, meningitis, leptomeningeal disease, diffuse axonal injury, and cortical ischemia [6]. Transient subcortical hypointensities on T2WI may also be seen in patients with status epilepticus and are often correlated with abnormal electroencephalographic activity localized to the affected lobes [6]. Recently, it was reported that transient subcortical hypointensity on T2WI may occur more frequently in encephalitis associated with myelin oligodendrocyte glycoprotein (MOG) antibody-associated disease compared to other causes of encephalitis [7].

There have been a few reports of the MRS findings about NKH [8]. In our patient, there was a decrease in NAA/Cre (0.98) and an increase in the lactate level in the left occipital lobe; the decreased NAA indicates neuronal damage or loss, whereas lactate is a substance found when anaerobic metabolism is increased. These findings indicate the occurrence of neuronal damage and disruption of normal metabolism on the affected side.

Furthermore, the patient demonstrated increased CBF on both ASL and ¹²³I-IMP SPECT. The poor visualization of cerebral veins on SWI suggests increased perfusion on the arterial side and enhanced oxygenation. ASL and ¹²³I-IMP SPECT are both techniques for measuring CBF; however, they possess distinct characteristics. ASL magnetically labels hydrogen atoms in arterial blood and measures CBF after a predetermined post-labeling delay (PLD). However, the arterial transit time varies among individuals and different brain regions, making it challenging to achieve accurate CBF evaluation without appropriate PLD settings [9]. In contrast, ¹²³I-IMP SPECT measures CBF in a fixed equilibrium state, rendering it less susceptible to imaging timing and enabling more stable assessments [10]. In this case, both ASL and ¹²³I-IMP SPECT confirmed increased blood flow in the left occipital lobe, clearly demonstrating the pathophysiological condition of increased CBF associated with NKH. Moreover, the clinical symptoms and abnormal MRI findings completely resolved following glycemic control, suggesting that these imaging findings were transient changes induced by hyperglycemia.

It has been speculated that the tendency to develop abnormalities in the occipital lobe may be related to dysfunction of the sympathetic nervous system [5]. Sympathetic stimulation increases cerebral vascular pressure in response to hemodynamic changes. However, the vertebrobasilar artery system, which supplies the occipital lobe, has limited autonomic regulatory capacity due to low sympathetic innervation. Diabetic patients often experience sympathetic autonomic dysfunction. In addition, they are prone to vascular endothelial dysfunction, disruption of the blood-brain barrier, and increased release of free radicals, all of which contribute to impaired blood flow regulation, particularly in the occipital lobe [5].

## Conclusions

This was a rare case of right homonymous hemianopsia and transient MRI abnormalities induced by NKH, which highlights the importance of recognizing NKH as a potentially reversible cause of acute visual disturbances in diabetic patients. Furthermore, advanced imaging techniques, including ASL, SPECT, and MRS, revealed reversible hemodynamic and metabolic changes, providing valuable insights into the underlying pathophysiological mechanisms. 

Clinically, these techniques not only confirmed the diagnosis but also demonstrated the potential for reversal of abnormal findings with appropriate treatment, highlighting their diagnostic and prognostic value. Moreover, in cases where typical MRI findings are not observed, comprehensive neuroimaging may enhance diagnostic accuracy, Further research is expected to elucidate the pathophysiology of this condition in greater detail, contributing to improved understanding and management.
